# Effect of Change in USMLE Step 1 Grading on Orthopaedic Surgery Applicants: A Survey of Orthopaedic Surgery Residency Program Directors

**DOI:** 10.5435/JAAOSGlobal-D-20-00216

**Published:** 2021-05-04

**Authors:** Alex Gu, Jacob Farrar, Safa C. Fassihi, Seth Stake, Pradip Ramamurti, Chapman Wei, Lauren E. Wessel, Duretti T. Fufa, Raj D. Rao

**Affiliations:** From the Department of Orthopaedic Surgery, George Washington University Hospital, Washington, DC (Dr. Gu, Dr. Farrar, Dr. Fassihi, Dr. Stake, Ramamurti, Wei, and Dr. Rao), and the Department of Hand Surgery, Hospital for Special Surgery, New York, NY (Dr. Wessel and Dr. Fufa).

## Abstract

**Introduction:**

Recently, the Federation of State Medical Boards and the National Board of Medical Examiners, cosponsors of the United States Medical Licensing Examination (USMLE), changed the USMLE Step 1 results from a three-digit score to a pass/fail format. The purpose of this study was to analyze the opinions of program directors (PDs) to predict how the evaluation of orthopaedic surgery residency applicants will change following the change.

**Methods:**

A 17-question online survey was distributed to PDs via e-mail. This survey covered program demographics, questions regarding the relative importance of various factors for selection of interviews, and perceived changes and effect of the scoring change. Responses were aggregated and analyzed.

**Results:**

PDs indicated that the three highest scored factors were (1) failure in prior attempts in USMLE/COMLEX examinations (4.7), (2) audition elective/rotation within your department (4.5), and (3) personal prior knowledge of the applicant (4.1). In addition, 38 PDs (81.1%) anticipate that they will require USMLE Step 2 clinical knowledge scores for interview consideration.

**Conclusion:**

Most orthopaedic surgery PDs think that the change in score reporting for the USMLE Step 1 will result in additional requirements and changes in how programs select applicants and do not support the decision.

The residency application has become increasingly challenging to navigate for both applicants and American College of Graduate Medical Education–accredited orthopaedic surgery residency programs. In 2018, 1192 applicants sent an average of 87.9 applications per applicant to American College of Graduate Medical Education–accredited orthopaedic surgery residencies.^1^ Approximately 100,000 applications were sent for 727 first-year orthopaedic surgery training positions,^1^ and each residency program received an average of 623 applications to fill 2 to 14 positions.^1^ The ratio of applications to residency positions for orthopaedic surgery is two standard deviations above the mean relative to other specialties, making it among the most competitive specialties.^2^

The increasing number of applications has followed the increasing popularity and competitiveness of the field over time. As with other competitive specialties, between 2006 and 2014, the number of available orthopaedic surgery positions increased at a rate of nine positions per year, whereas the number of applicants increased by 65 per year.^3^ The standardized Step 1 scores from the United States Medical Licensing Examination (USMLE) have previously been found to be very important in the selection of residency applicants in competitive specialties such as orthopaedic surgery, dermatology, and urology.^4,5,6,7,8^ In fact, a previous study reported that 83% of program directors (PDs) used minimum USMLE Step 1 scores as a screening tool to decrease the number of applications necessary for review,^4^ and with increasing numbers of applicants, programs have been using progressively higher minimum scores to screen their applicant pools.^4,5,6,7,8^ By polling United States orthopaedic surgery PDs, Schrock et al.^4^ found that 78% of programs required a USMLE Step 1 score of ≥210, 75% required a score ≥220, 53% required a score ≥230, and 21% required a score ≥240. Furthermore, the mean USMLE Step 1 score for matched applicants in orthopaedic surgery (248) trailed only plastic surgery (249) and dermatology (249).^9^ These data confirm the strong role of the numeric USMLE Step 1 scores in the screening and ranking of residency applicants to orthopaedic surgery.

In addition to being a useful tool for programs during the selection process, USMLE Step 1 scores have historically aided medical students in determining their competitiveness as applicants.^10,11^ Because of the selectivity of orthopaedic surgery, knowledge of how applications are processed is important for applicants to determine not only their competitiveness but also how to best optimize their selected pool of programs to which they intend to apply.

On February 12, 2020, the Federation of State Medical Boards and the National Board of Medical Examiners, cosponsors of the USMLE, announced that the USMLE Step 1 results would be reported as pass/fail rather than as a three-digit score.^12^ There have been no published data about how orthopaedic surgery PDs will assess and rank orthopaedic surgery applicants without a numeric USMLE Step 1 score. The purpose of this study was to analyze the opinions of PDs across the country to predict how the screening of applications will change following the change in USMLE Step 1 score format. We hypothesize that there will be a greater emphasis placed on USMLE Step 2 clinical knowledge (CK) and away rotation performance given the lack of other standardized objective data to compare students across different medical schools.

## Methods

### Survey Population

Following institutional review board approval, an online survey (Qualtrics Experience Management) was used to query orthopaedic surgery residency PDs in March and April 2020. A link to the survey was sent to the PDs' e-mail addresses. E-mail addresses were determined using a combination of those available through the American Medical Association Fellowship and Residency Electronic Interactive Database (FRIEDA) database, the American Academy of Orthopaedic Surgeons member database, and institution-specific websites. If an e-mail address could not be identified, the institution's program coordinator was then contacted to acquire the PD's e-mail address. All programs were identified using the latest listing of programs on the Electronic Residency Application Service for the 2019 to 2020 cycle.^13^

Publicly available historical data from the annual PD survey produced by the National Residency Matching Program (NRMP) and the Association of American Medical Colleges were used to provide baseline metrics.^14^ In this survey, PDs are asked to indicate factors they use in selecting applicants for interview and to rate the importance of each factor. The most recently available 2018-year data, before the Step 1 score grading change, were used to provide a baseline for comparison with our data. In the NRMP survey, PDs are asked to rate the importance of each factor on a scale of 1 to 5, which we mimicked in our survey. In total, 47 orthopaedic surgery PDs contributed to the 2018 NRMP survey.^14^

### Survey Content

The online survey included 17 questions, with four questions related to demographics and characteristics of the program, including description of program, location of program, size of program, and optional PD name. One block of questions inquired into the PDs' level of anticipated importance of other factors that may be used in evaluation of applicants in the absence of a numeric USMLE Step 1 score, with a visual analog scale from 1 to 5, which reflects the format of the NRMP importance rating scale.^14^ Additional questionnaire items in our study reviewed the current and future utility of USMLE Step 1 and Step 2 CK results, the importance of other application factors, and the effect of the change in USMLE Step 1 score format on applicant well-being (Appendix 1, http://links.lww.com/JG9/A128).

Survey responses were all anonymous after removal of PD name if provided and aggregated for analysis. Descriptive statistics were used to characterize demographic data. The previously reported mean importance of the USMLE Step 1 from the 2018 PD survey responses was 4.1, and the average anticipated importance of USMLE Step 1 considering its transition to pass/fail was 3.3.^14^ These two points were used as baselines for comparison to mitigate potential bias from visual analog scale utilization.^15,16^ In this fashion, the differences from the averages were used instead of absolute differences between the two values for our analysis.

## Results

### Study Cohort

In total, 53 of 193 (27.4%) orthopaedic surgery PDs responded to the survey during the study period (Table 1). Most programs were university based (66.0%), located in the mid-Atlantic location of the United States (22.6%) and had between 4 to 8 postgraduate year-1 positions (64.2%) in 2020. The remaining descriptions of programs are further listed in Table 1.

**Table 1:** Demographics of Surveyed Program Respondents

Category	Number (n)	Percentage (%)
Program description		
University based	35	66.0
Community based	5	9.4
Community based/university affiliated	12	22.6
Region		
New England	1	1.9
Mid-Atlantic	5	9.4
East North Central	12	22.6
West North Central	11	20.8
South Atlantic	6	11.3
South West	6	11.3
East South Central	6	11.3
Rocky Mountain region	0	0.0
West Pacific	4	7.5
Total PGY-1 positions	3	5.7
1-3 positions		
4-8 positions	18	34.0
9+ positions	34	64.2

PGY = postgraduate year

New England: CT, MA, ME, NH, RI, and VT; Mid-Atlantic: NJ, NY, and PA; East North Central: IL, IN, MI, OH, and WI; West North Central: IA, KS, MN, MO, ND, NE, and SD; South Atlantic: DC, DE, FL, GA, MD, NC, SC, VA, and WV; South West: TX, AZ, OK, and NM; East South Central: AL, KY, MS, TN, AR, LA, and PR; Rocky Mountain Region: MT, ID, WY, UT, CO, and NV; West Pacific: WA, OR, CA, HI, and AK.

### Importance of Specific Factors

**Figure 1:**
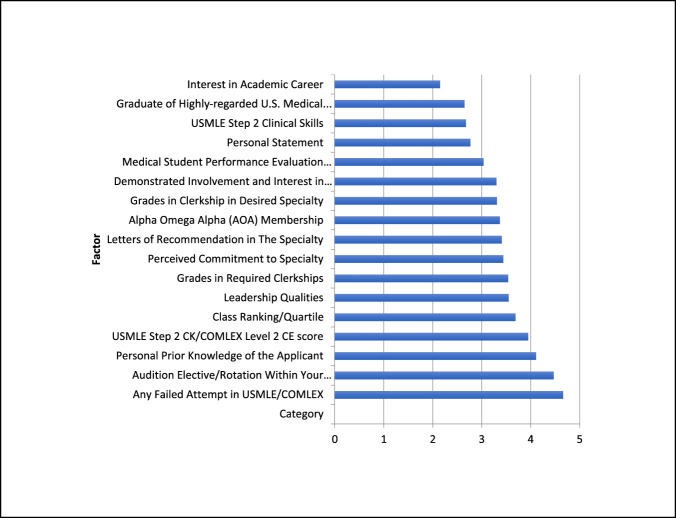
Graph showing anticipated importance of various factors for reviewing orthopaedic surgery residency applicants. USMLE =United States Medical Licensing Examination

**Figure 2:**
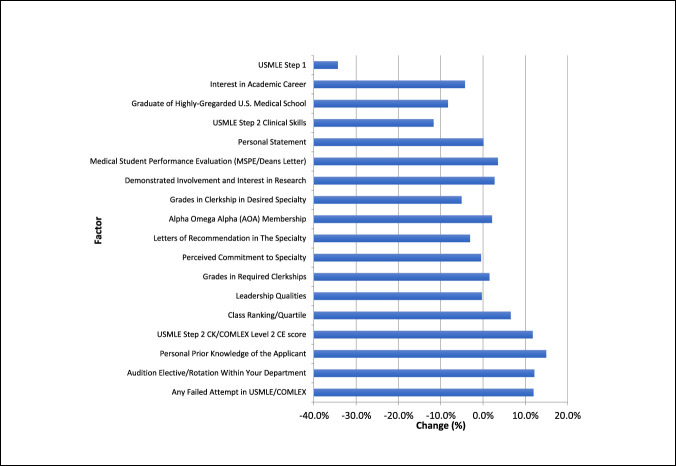
Graph showing anticipated change of importance of various factors for reviewing orthopaedic surgery residency applicants following grading change. USMLE =United States Medical Licensing Examination

When asked about the anticipated importance of USMLE Step 1 outcome following conversion to pass/fail reporting, the average score was 2.1. After adjustment, there was a 34.3% decrease in relative importance compared with responses in 2018. In the absence of a numeric USMLE Step 1 score, the five factors receiving the highest scores for anticipated importance in reviewing orthopaedic residency applications were (1) failure in prior attempts in USMLE/Comprehensive Osteopathic Medical Licensing Examination (COMLEX) (4.7) examinations, (2) audition elective/rotation within your department (4.5), (3) personal prior knowledge of the applicant (4.1), (4) USMLE Step 2 CK/COMLEX Level 2 CE Score (4.0), and (5) class ranking/quartile (3.7). Compared with 2018 responses, there was an increase in relative importance of all five factors (Figures 1 and 2).

### USMLE Step 1 and Step 2 CK

Among survey responses, 36 of 53 respondents (65.8%) considered USMLE Step 1, in its current, numerically scored format, to be either very or extremely important when selecting applicants for interview (Table 2). In contrast, only six respondents (11.3%) anticipated viewing USMLE Step 1 as very or extremely important after conversion to pass/fail scoring. Currently, 19 of 53 programs (35.8%) require a USMLE Step 2 CK score for interview consideration (Table 2). Following the scoring change, 43 PDs (81.1%) anticipate that they will require USMLE Step 2 CK for interview consideration (Table 2).

**Table 2:** Questions Regarding USMLE Step 1 and Step 2 CK

Question	Not at All Important		Slightly Important		Moderately Important		Very Important		Extremely Important	
	n	%	n	%	n	%	n	%	n	%
Importance of USMLE Step 1 for selecting interview applicants when listed as a 3-digit score	0	0.0	7	13.2	10	18.9	20	37.7	16	30.2
Anticipated importance of USMLE Step 1 for selecting interview applicants when listed as pass/fail	26	49.1	15	28.3	6	11.3	5	9.4	1	1.9
	Yes		No							
	n	%	n	%						
No. of programs currently requiring Step 2 CK for interview	19	35.8	34	64.2						
	Definitely Not		Probably Not		Might or Might Not		Probably Yes		Definitely Yes	
	n	%	n	%	n	%	n	%	n	%
Anticipated potential for requiring Step 2 CK for interview	0	0.0	3	5.7	7	13.2	12	22.6	31	58.5

CK = clinical knowledge, USMLE = United States Medical Licensing Examination

### Effect on Applicants

When asked whether the score change would affect their ability to recruit competitive applicants, most PDs responded neutrally (Table 3). However, most PDs responded negatively when asked whether the score change would improve diversity among their incoming resident class, with 18.9% responding “Definitely Not” and 47.2% responding “Probably Not.” When asked whether the USMLE Step 1 scoring change would decrease the likelihood of applicants from lower-ranked medical schools being selected for an interview, 37.7% of PDs responded either “Probably Yes” or “Definitely Yes” (Table 3). Most surveyed PDs did not think that changes in USMLE Step 1 score reporting would negatively affect lower socioeconomic classes or international applicants (Table 3).

**Table 3:** Effect of USMLE Step 1 Score Reporting on Application Process

Question	Definitely Not		Probably Not		Might or might not		Probably Yes		Definitely Yes	
	n	%	n	%	n	%	n	%	n	%
Ability to recruit competitive applicants	7	13.2	13	24.5	20	37.7	3	5.7	10	18.9
Improve diversity of applicants	10	18.9	25	47.2	14	26.4	4	7.5	0	0.0
Decreased likelihood of applicants from lower-ranked school being selected for interviews	5	9.4	15	28.3	13	24.5	10	18.9	10	18.9
Decreased likelihood of applicants from international graduate schools being selected for an interview	14	26.4	6	11.3	6	11.3	13	24.5	14	26.4
Decreased likelihood of applicants from lower socioeconomic status being selected for interviews	14	26.4	21	39.6	14	26.4	4	7.5	0	0.0
Improve mental health or wellness in medical school	8	15.1	17	32.1	18	34.0	10	18.9	0	0.0
Do you support this decision to make USMLE Step 1 pass/fail	33	62.3	7	13.2	7	13.2	3	5.7	3	5.7

USMLE = United States Medical Licensing Examination

### Mental Health and Support

When asked whether the change in USMLE Step 1 scoring would improve mental health or wellness among medical students, most surveyed PDs stated either probably not (17/53; 32.1%) or might or might not (18/53; 33.9%; Table 3). When asked whether they supported the decision to change USMLE Step 1 grading to pass/fail, 40 orthopaedic surgery PDs (75.5%) said that they definitely did not or probably did not support this decision (Table 3).

### Additional Considerations

**Figure 3:**
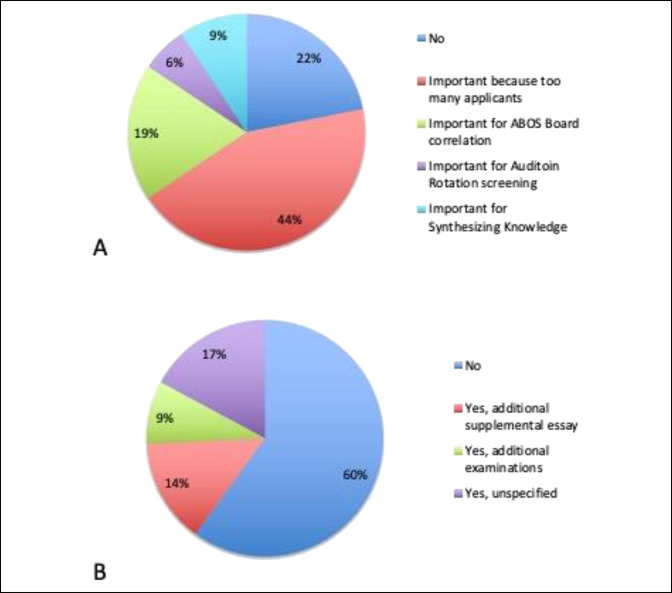
**A**, Responses to if PDs thought that Step 1 is important and (**B**) if PDs thought that additional requirements for applications would be required. PD = program director

When asked whether orthopaedic surgery as a field considered USMLE Step 1 examinations differently than other fields, most respondents said yes (78%; Figure 3). Among those who said yes, most said that the examination was important as a screening tool primarily because there are too many applicants (Figure 3). When asked whether they anticipated any additional requirements other than USMLE Step 2 CK, most (60%) said “no.” Among the remaining who said “yes,” additional suggestions included adding a supplemental essay or additional examinations (Figure 3).

## Discussion

Our data demonstrate that the implementation of a pass/fail scoring system will substantially affect and alter the orthopaedic surgery resident selection process. With the proposed change to pass/fail, the USMLE Step 1 score decreases in relevance. Instead, audition rotations, USMLE Step 2 CK, and prior knowledge of the applicant will likely be considered the most important factors in evaluating candidates for an orthopaedic surgery residency interview invitation.

With the transition of the USMLE Step 1 examination to a pass/fail model, applicants should clearly understand factors in their application that residency programs will be most closely evaluating. Before this announcement by the Federation of State Medical Board and NBME, Chen et al.^2^ reviewed characteristics of those applicants who successfully matched into orthopaedic surgery residency. Among these factors, USMLE Step 1 scores, research productivity, and Alpha Omega Alpha (AOA) membership were the strongest predictors of interview yield, with AOA membership having the greatest influence. This study also showed trends suggesting that the USMLE Step 1 score was used primarily as a screening tool. Applicants with a USMLE Step 1 score ≥ 240 were 10% more likely to receive an interview offer than applicants with a USMLE Step 1 score < 240 (*P* < 0.05). However, no significant difference was found between applicants with a score of 240 to 250 compared with those with scores of 250 to 260 or >260. Class rank was also significantly associated with higher interview yield, with applicants in the top third of the class receiving 8% more interviews than those in the middle and bottom third.^2^ Schrock et al.^3^ retrospectively reviewed match statistics from 2006 to 2014, finding that USMLE Step 1 scores (*P* < 0.001), USMLE Step 2 CK scores (*P* < 0.01), mean number of research products (*P* = 0.035), and AOA membership (*P* < 0.001) were all significant predictors of a successful match. This study also found that a higher proportion of successful applicants attended a top-40, NIH-funded medical school (*P* < 0.001).^3^ Overall, USMLE Step 1 scores, in addition to USMLE Step 2 scores, AOA membership, and research involvement appear to be critical factors in the interview selection process.

Our study found that PDs will place an increased emphasis on Step 2 CK scores in the absence of a standardized USMLE Step 1 score. Historically, the USMLE Step 2 CK examination was taken during the last year of medical education and played a variable and often minor role in the orthopaedic surgery residency application process.^4^ The present study found that 81.1% of PDs will require USMLE Step 2 CK scores following the change in USMLE Step 1 scoring, versus the 11.3% of PDs who required USMLE Step 2 CK when the USMLE Step 1 examination was numerically scored. Notably, a high score on the USMLE Step 2 examination could previously be used by students to offset a poor score on the USMLE Step 1 examination. With USMLE Step 2 CK now acting as the lone standardized measure to compare applicants, performance on the examination may not only be more closely scrutinized, but there would be no subsequent USMLE examination for the applicant to demonstrate improvement in the case of poor performance on USMLE Step 2 CK.

As the term implies, “standardized testing” is intended to provide an objective standard with which students from a variety of different institutions and backgrounds can be evaluated in a consistent manner. The USMLE Step 1 examination provided a means by which students with varying levels of institutional resources could have an equal or near-equal opportunity to demonstrate their comprehension of medical facts and their ability and desire to prepare for these tests. Our study demonstrated a desire on the part of orthopaedic surgery PDs to continue to have a form of standardized evaluation, with a shift to using USMLE Step 2 CK scores following conversion of USMLE Step 1 scoring to pass/fail. More subjective criteria, such as subinternship/audition rotations and personal knowledge of applications, will also receive greater weight, potentially creating regional barriers. The longer term consequences of any reduction in heterogeneity of the applicant pool will need to be reassessed over time.

Prior work by O'Donnell et al.^17^ found that while rotating at a program increased an applicant's odds of matching there by a factor of 1.5, no correlation was found between the total number of external (visiting) orthopaedic rotations performed and the likelihood of matching. In that study, 84% of PDs report that audition rotations at outside institutions were viewed as neither positive nor negative factors. Following the change in USMLE Step 1 score reporting, our study found a 12% increase in the importance of audition rotations and a 15% increase in the importance of personal prior knowledge of the applicant. With the change in Step 1 result format, 40% of PDs also stated that they would be less likely to offer an interview to an applicant hailing from a lower-ranked medical school. Applicants will almost certainly feel the need to apply to more programs or partake in more audition rotations, both of which can place a significant financial burden on students who are often already in substantial debt from student loans.^18^ Both candidates and programs should carefully consider the economic and emotional strain of planning for and completing multiple subinternships. Although the switch to a pass/fail grade for USMLE Step 1 may have been precipitated in part by the desire to improve the well-being of medical students, further studies are needed to determine whether the unintended consequences of this switch may, in fact, be more detrimental to the overall well-being of medical students.

Although the present study did not find that PDs expected any different treatment of applicants from lower socioeconomic statuses, the application process is costly for candidates. The average medical student graduates with nearly $200,000 debt.^19^ This burden is increased substantially by residency interviews, with the average applicant spending approximately $7000 on the interview process and 72% of applicants having to borrow money to pay for these expenses.^2^ Camp et al.^18^ prospectively evaluated the cost of the application process, finding the average cost of the application itself to be as great as $5000 and the average cost of interviewing to be as great as $20,000. As previously mentioned, medical students may choose to complete more audition rotations with the elimination of numeric USMLE Step 1 scoring, driving up the cost of their medical education due to housing and travel. These additional expenses may have a detrimental effect on the application process and competitiveness in the orthopaedic surgery application process for students with lower socioeconomic means.

The present study does have limitations. First, it should be noted that the results of our survey are based merely on the predictions by PDs and that none of these factors have been correlated with actual match results. In addition, the survey response rate was 27% and thus reflects a minority of the nation's PD population, thus reducing the external validity of this analysis. Despite this limited representation, the response rate is nearly identical to recent, similarly designed studies in other specialties,^20^ and similar to the 2018 NRMP Match data response rate (47/179 = 26%).^14^ Another source of potential bias could be the demographic distribution of the survey respondents. Although the 2018 NRMP Match data do not disclose demographic data associated with its respondents, the current study's results paralleled the national distribution of programs. Most respondents were from university-based programs (66.0%), similar to the national rate of 52.8%.^21^ In addition, our rate of community-based programs (9.4%) and community-based/university affiliated programs (22.6%) mirror the nationwide distribution of 12.7% and 30.5%, respectively.^21^ Although the geographic classification does not match the national spread, it is unlikely that geography affected the importance of the USMLE Step 1 score in evaluating applicants. Finally, as many of the goals of transitioning the USMLE Step 1 to pass/fail scoring are applicant centric, it would be of great interest to survey orthopaedic surgery–inclined medical students as to their own impressions of the effect of this change on the competitiveness of their application. One of the fundamental aims for converting USMLE Step 1 scoring to pass/fail was to reduce emotional anxiety related to test taking. Future studies examining medical students' emotions and perceptions regarding these changes are necessary to fully evaluate the effect of this change on their overall well-being.

## Conclusion

Most orthopaedic surgery PDs think that the change in score reporting for the USMLE Step 1 will result in additional requirements and changes in how programs select applicants. Furthermore, most surveyed orthopaedic surgery PDs did not think that this change will benefit the mental well-being of orthopaedic surgery candidates and ultimately did not support the decision to change USMLE Step 1 to pass/fail.
